# The role of plasma neurofilament light chain and glial fibrillary acidic protein in subjective cognitive decline and mild cognitive impairment

**DOI:** 10.1007/s10072-023-07065-4

**Published:** 2023-09-19

**Authors:** Salvatore Mazzeo, Assunta Ingannato, Giulia Giacomucci, Silvia Bagnoli, Arianna Cavaliere, Valentina Moschini, Juri Balestrini, Carmen Morinelli, Giulia Galdo, Filippo Emiliani, Diletta Piazzesi, Chiara Crucitti, Daniele Frigerio, Cristina Polito, Valentina Berti, Sonia Padiglioni, Sandro Sorbi, Benedetta Nacmias, Valentina Bessi

**Affiliations:** 1 Department of Neuroscience, Psychology, Drug Research and Child Health, University of Florence, Florence, Italy; 2 Research and Innovation Centre for Dementia-CRIDEM, Azienda Ospedaliero-Universitaria Careggi, Florence, Italy; 3 IRCCS Fondazione Don Carlo Gnocchi, Florence, Italy; 4 Department of Biomedical, Experimental and Clinical Sciences “Mario Serio”, University of Florence, 50134 Florence, Italy; 5 Regional Referral Centre for Relational Criticalities- 50139, Tuscany Region, Italy

**Keywords:** Glial fibrillary acidic protein, Neurofilament light chain, Alzheimer’s disease, Mild cognitive impairment, Subjective cognitive decline, Biomarkers

## Abstract

**Introduction and aim:**

NfL and GFAP are promising blood-based biomarkers for Alzheimer's disease. However, few studies have explored plasma GFAP in the prodromal and preclinical stages of AD. In our cross-sectional study, our aim is to investigate the role of these biomarkers in the earliest stages of AD.

**Materials and methods:**

We enrolled 40 patients (11 SCD, 21 MCI, 8 AD dementia). All patients underwent neurological and neuropsychological examinations, analysis of CSF biomarkers (Aβ_42_, Aβ_42_/Aβ_40_, p-tau, t-tau), Apolipoprotein E (*APOE*) genotype analysis and measurement of plasma GFAP and NfL concentrations. Patients were categorized according to the ATN system as follows: normal AD biomarkers (NB), carriers of non-Alzheimer's pathology (non-AD), prodromal AD, or AD with dementia (AD-D).

**Results:**

GFAP was lower in NB compared to prodromal AD (*p* = 0.003, *d* = 1.463) and AD-D (*p* = 0.002, *d* = 1.695). NfL was lower in NB patients than in AD-D (*p* = 0.011, *d* = 1.474). NfL demonstrated fair accuracy (AUC = 0.718) in differentiating between NB and prodromal AD, with a cut-off value of 11.65 pg/mL. GFAP showed excellent accuracy in differentiating NB from prodromal AD (AUC = 0.901) with a cut-off level of 198.13 pg/mL.

**Conclusions:**

GFAP exhibited excellent accuracy in distinguishing patients with normal CSF biomarkers from those with prodromal AD. Our results support the use of this peripheral biomarker for detecting AD in patients with subjective and objective cognitive decline.

## Introduction 

Research and clinical practice on Alzheimer's disease (AD) are at a turning point. The Food and Drug Administration (FDA) has provisionally approved two anti-amyloid monoclonal antibodies for the treatment of patients with mild cognitive impairment (MCI) due to AD and mild AD dementia [1]. This represents a significant milestone as these are the first disease-modifying therapies for AD. Consequently, neurologists, researchers, and health services will face increasing demands for diagnostic assessments of patients with cognitive disorders, as well as the need to diagnose AD at its earliest stages to halt the pathological process before neurodegeneration begins [2]. Currently used disease biomarkers, such as cerebrospinal fluid (CSF) biomarkers [3, 4], PET neuroimaging, and brain MRI [5–7], are highly accurate in detecting AD pathology. However, their application on large populations is severely limited due to cost, limited accessibility, and invasiveness.

Blood-based biomarkers are considered promising tools that could overcome these limitations and enable biomarker assessments even at the primary care level, where most individuals with cognitive symptoms present [8]. Among the peripheral biomarkers, Neurofilament light chain (NfL) and Glial fibrillary acidic protein (GFAP) are emerging as reliable biomarkers of AD, being associated with CSF biomarkers [9–11]. NfL is a component of the neuronal cytoskeleton and is released into the cerebrospinal fluid and blood following neuronal damage [12]. GFAP is a marker of reactive astrogliosis [13], associated with morphological, molecular, and functional remodeling of astrocytes surrounding Aβ plaques [14]. Elevated levels of NfL and GFAP have been observed in subjective cognitive decline (SCD), MCI and AD dementia [15–17]. However, the accuracy of plasma NfL and GFAP in predicting underlying AD pathology in patients with SCD and MCI has been poorly explored so far [10, 18–20]. Therefore, our aim is to assess the accuracy of plasma NfL and GFAP in predicting AD pathology in SCD and MCI patients.

## Materials and Methods

### Study design, patients, and assessments

This is a cross-sectional analysis involving 40 consecutive patients (11 patients with SCD, 21 patients with MCI, and 8 patients with AD dementia) referred to the Centre for Alzheimer’s Disease and Adult Cognitive Disorders of Careggi Hospital in Florence for assessment of cognitive decline, since July 2018 to November 2022. We included patients that met the following criteria: (1) clinical diagnosis of AD dementia [21], (2) clinical diagnosis of MCI [22], (3) clinical diagnosis of SCD [23]. Exclusion criteria were: history of head injury, current systemic and/or neurological disease other than AD, major depression, or substance use disorder. All patients underwent: comprehensive clinical assessment, neurological examination; extensive neuropsychological investigation and brain MRI or CT scan; ^18^F-Fluorodeoxyglucose-PET brain scan (^18^F-FDG-PET); blood collection for Apolipoprotein E (*APOE)* genotype analysis and measurement of plasma NfL and GFAP concentration; CSF collection for Aβ_42_, Aβ_42_/Aβ_40_, total-tau (t-tau) and phosphorylated-tau (p-tau) measurement. We defined age at baseline as the age at the time of plasma collection, disease duration as the time from the onset of symptoms to baseline examination, and positive family history of dementia if one or more first-degree relatives were reported to have documented cognitive decline. We defined disease duration as the timeframe from the onset of symptoms to baseline examination and family positive history of dementia if one or more first-degree relatives were reported to have documented cognitive decline.

### Neuropsychological assessment

All subjects were evaluated by an extensive neuropsychological battery including: global cognition measure (MMSE) [24], tasks exploring verbal and spatial short- working and long-term memory (Digit and Visuo-spatial Span forward and backward [25], Rey Auditory Verbal Learning Test – RAVLT [26], Short Story Immediate and Delayed Recall [27], Rey-Osterrieth complex figure recall [28]), attention (Trail Making Test A, attentional matrices [29]), language (Category Fluency Task [30] and Phonemic Fluency Task [26]), constructional praxis (Rey-Osterrieth complex figure copy) and executive function (Trail Making Test B [31], Stroop Test [32]). In SCD patients, cognitive complaints were explored at baseline using a survey based on the Memory Assessment Clinics-Questionnaire [33].

### Plasma GFAP and NfL analysis

Blood was collected by venipuncture into standard polypropylene EDTA test tubes (Sarstedt, Nümbrecht, Germany) and centrifuged within two hours at 1300 rcf at room temperature for 10 min. Plasma was isolated and stored at -80 °C until testing. Plasma biomarkers analysis was performed with Simoa NF-Light SR-X kit (cat. No 103400) and with Simoa GFAP SR-X kit (cat. No 102336) for human samples provided by Quanterix Corporation (Lexington, MA, USA) on the automatized Simoa SR-X platform (GBIO, Hangzhou, China), following the manufacturer’s instructions [34]. The Lower Limit of Quantification (LLOQ) and the Limit of Detection (LOD) provided by the NF-Light kit were 0.316 pg/mL and 0.0552 pg/mL, respectively. The LLOQ and the LOD of GFAP kit were 1.37 pg/mL and 0.26 pg/mL, respectively. Plasma NfL and GFAP concentrations of all samples were detected in a single run basis. Quality controls (low NfL concentration = 5.08 pg/mL and high NfL concentration = 169 pg/mL; low GFAP concentration = 48.0 pg/mL and high GFAP concentration = 1063 pg/mL) were included in the array and tested with samples. A calibration curve was determined from measurements of serially diluted calibrators provided by Quanterix. Plasma samples and controls were diluted at a 1:4 ratio and measured in duplicate with calibrators.

### APOE ε4 genotyping

A standard automated method (QIAcube, QIAGEN) was used to isolate DNA from peripheral blood samples. *APOE* genotypes were investigated by high-resolution melting analysis (HRMA)[35]. Two sets of PCR primers were designed to amplify the regions encompassing rs7412 [NC_000019.9:g[M13] [GG14] 0.45412079C > T] and rs429358 (NC_000019.9:g.45411941 T > C). The samples with known *APOE* genotypes, which had been validated by DNA sequencing, were used as standard references.

### CSF Collection and biomarkers analysis

CSF was collected at 8.00 a.m. by lumbar puncture, immediately centrifuged and stored at -80 °C until performing the analysis. Aβ_42_, Aβ_42_/ Aβ_40_ ratio, t-tau, and p-tau were measured using a chemiluminescent enzyme immunoassay (CLEIA) analyzer LUMIPULSE G600 (Fujirebio). Cut-offs for normal values were: for Aβ_42_ > 670 pg/ml, Aβ_42_/Aβ_40_ ratio > 0.062, t-tau < 400 pg/ml and p-tau < 60 pg/ml [36].

### Brain ^18^F-FDG-PET acquisition and rating

^18^F-FDG-PET scans were acquired following the EANM procedure guidelines [37], using an advanced hybrid PET-CT scanner in 3D list mode. PET data were reconstructed using 3D iterative algorithm, corrected for attenuation, random and scatter using the manufacturer’s software. A trained nuclear medicine physician visually rated all scans as positive or negative, according to the European Association of Nuclear Medicine and European Academy of Neurology recommendations [38], as described in a previous work [39].

### Classification of patients according to the ATN classification

Based on biomarker results, patients were classified according to the NIA-AA Research Framework [40]: patients were rated as A + if at least one of the amyloid biomarkers (Aβ_42_ or Aβ_42_/ Aβ_40_ ratio) revealed the presence of Aβ pathology, and as A- if none of the biomarkers revealed the presence of Aβ pathology. Patients were classified as T + or T- if CSF p-tau concentrations were higher or lower than the cut-off value, respectively. Patients were classified as N + if at least one neurodegeneration biomarker was positive (CSF t-tau higher than the cut-off value or positive ^18^F-FDG-PET). In the case of discordant results between CSF and ^18^F-FDG-PET, we considered only the pathologic result. Based on this first classification, considering our sample size and to avoid creating excessively small groups, we classified patients as follows: i) carriers of normal biomarkers (NB) if all the biomarkers were negative (A-/T-/N-); ii) non Alzheimer’s pathologic change (non-AD) if they had positive p-tau and/or t-tau (A-/T + /N-, A-/T + /N-, A-/T + /N +); iii) prodromal AD (SCD and MCI) or AD-dementia (AD) if they had positive amyloid biomarkers and positive p-tau; we included in this group also patients with isolated Aβ pathology (A + /T-/N-, A + /T + /N-, A + /T + /N +).

### Statistical analysis

All statistical analyses were performed via IBM SPSS Statistics Software Version 25 (SPSS Inc., Chicago, USA) and the computing environment R 4.2.3 (R Foundation for Statistical Computing, Vienna, 2013). Figures were created using R 4.2.3 and Adobe Illustrator (Adobe Inc., San Jose, California). Statistical significance received Bonferroni adjustment for multiple comparisons being accepted at *p* < 0.005. Distributions of all variables were assessed through the Shapiro–Wilk test. As NfL and GFAP were not normally distributed, we applied a log10 transformation. This transformation resulted in normally distributed data that met the assumptions of parametric statistical tests that were necessary to evaluate our hypotheses. We conducted descriptive statistics to examine the central tendency and variability of our data using means and standard deviations (SD) for continuous variables and frequencies or percentages and 95% confidence interval (95% CI) for categorical variables, respectively. We used t-test for comparison between two groups, one-way analysis of variance (ANOVA) with Bonferroni post-hoc test for comparison between three or more groups, Pearson’s correlation coefficient to evaluate correlations between groups’ numeric measures and chi-square test to compare categorical data. To adjust for possible confounding factors, we used analysis of covariance (ANCOVA). We calculated the size effect by Cohen’s *d* for normally distributed numeric measures, η^2^ for ANOVA and Cramer’s V for categorical data. We constructed receiver operating characteristic (ROC) curves and calculated the area under the curve (AUC) to evaluate the performance of plasma NfL and GFAP to predict AD. We used the maximize metric method to determine the optimal cut-off value for NfL and GFAP and calculated accuracy, sensitivity, and specificity.

## Results

### Description of the groups

Sixteen patients (40.00%, 8 SCD and 8 MCI) were classified as NB, four patients (10.00%, 1 SCD and 3 MCI) were rated as non-AD, 12 patients (30.00%, 2 SCD and 10 MCI) were rated as prodromal AD, including three A + /T-/N-. All eight patients with clinical diagnosis of AD showed biomarkers consistent with AD (6 A + /T + /N + and 2 A + /T-/N +) and were defined as AD-dementia. Thirteen patients (32.50%) had positive ^18^F-FDG-PET, including five patients with prodromal AD (1 SCD and 4 MCI) and all the eight AD-dementia patients. Negative ^18^F-FDG-PET scans showed normal brain metabolism and were not suggestive for any other neurological conditions. A detailed description of distributions of A/T/N subtypes among groups (SCD, MCI and AD-dementia) is reported in Table 1.

**Table 1:** Distributions of A/T/N subgroups among diagnosis groups

	SCD	MCI	AD dementia
NB	8 A-/T-/N-	8 A-/T-/N-	0
non-AD	1 A-/T + /N +	1 A-/T-/N + 2 A-/T + /N +	0
prodromal AD/ AD-dementia	2 A + /T + /N +	2 A + /T-/N- 8 A + /T + /N +	2 A + /T-/N + 6 A + /T + /N +

Patients were rated as:

A + if at least one of the amyloid biomarkers (Aβ_42_ or Aβ_42_/ Aβ_40_ ratio) revealed the presence of Aβ pathology, or A- if none of the biomarkers revealed the presence of Aβ pathology;

T + or T- if CSF p-tau concentrations were higher or lower than the cut-off value, respectively;

N + if at least one neurodegeneration biomarker was positive (CSF t-tau higher than the cut-off value or positive ^18^F-FDG-PET) or N- if none of the biomarkers revealed neurodegeneration. In the case of discordant results between CSF and ^18^F-FDG-PET, we considered only the pathologic result.

Patients in the NB group were younger than non-AD (*p* = 0.001, *d* = 2.448) and had higher MMSE compared to prodromal AD (*p* = 0.002, *d* = 1.576) and AD-dementia (*p* < 0.001, *d* = 2.042). There were no differences in gender, years of education or *APOE* frequencies between NB, non-AD, prodromal AD and AD-dementia (Table 2).

**Table 2:** Comparisons between groups

	NB	non-AD	prodromal AD	AD-dementia
N	16	4	12	8
Age, years	62.51(8.33) ^**a**^	78.64 (4.11) ^**a**^	70.94 (5.65)	71.64 (3.51)
Years of education	12.40 (3.64)	10.75 (6.07)	13.25 (3.41)	12.83 (4.26)
MMSE	27.09 (2.14) ^**b,c**^	27.12 (1.64)	23.39 (2.96) ^**b**^	22.26 (2.20) ^**c**^
Gender, female	11 (68.75%)	3 (75.00%)	6/6 (50.00%)	4 (50.00%)
*APOE* ε4^+^	6 (40.00%)	1 (25.00%)	5 (45.00%)	2 (29.00%)
Aβ_42_ (pg/mL)	1075.62 (245.24) ^**d,e**^	1408.00 (625.81)^**f, g**^	568.66 (138.17)^**d, f**^	460.37 (116.78)^**e, g**^
Aβ_42_/Aβ_40_,	0.097 (0.01) ^**h,i**^	0.082 (0.021)^**j, k**^	0.049 (0.016)^**h, j**^	0.050 (0.01) ^**i, k**^
p-tau (pg/mL)	31.12 (13.11) ^**l, m**^	73.90 (20.73)	105.56 (70.39)^**l**^	107.37 (37.05)^**m**^
t-tau (pg/mL)	250.31 (94.37) ^**n, o**^	537.00 (104.57)	646.48 (390.37)^**n**^	683.50 (175.57)^**o**^
LogNfL (pg/mL)	0.99 (0.14) ^**p**^	1.23 (0.06)	1.15 (0.20)	1.28 (0.28)^**p**^
LogGFAP (pg/mL)	2.05 (0.21) ^**q, r**^	2.37 (0.26)	2.38 (0.20) ^**q**^	2.43 (0.24) ^**r**^

Values quoted in the table are mean (SD) for continuous variables and frequencies (percentages) for dichotomic variables. Between-groups comparisons: ANOVA with Bonferroni post-hoc. Categorical data comparisons: χ^2^ test. Size effect: Cohen’s *d* for continuous measures, Cramer’s V for categorical data. Statistical significance received adjustment for multiple comparisons being accepted at p < 0.005

^**a**^ *p* = 0.001, *d* = 2.448; ^**b**^ *p* = 0.002, *d* = 1.576; ^**c**^ *p* < 0.001, *d* = 2.829; ^**d**^ *p* < 0.001, *d* = 1.901; ^**e**^ *p* < 0.001, *d* = 2.39;^**f**^ *p* < 0.001, *d* = 3.193; ^**g**^ *p* < 0.001, *d* = 3.684; ^**h**^ *p* < 0.001, *d* = 3.330; ^**i**^ *p* < 0.001, *d* = 3.269;^**j**^ *p* < 0.001, *d* = 2.706; ^**k**^ *p* < 0.001, *d* = 2.645; ^**l**^ *p* < 0.001, *d* = 1.689; ^**m**^ *p* = 0.002, d 0 1.731; ^**n**^ *p* < 0.001, *d* = 1.658; ^**o**^ *p* < 0.001, *d* = 1.812; *p* = 0.011, *d* = 1.474; ^**p**^ *p* = 0.004, *d* = 1.474; ^**q**^ *p* = 0.003, *d* = 1.463; ^**r**^ *p* = 0.002, *d* = 1.695

### Comparisons of CSF and blood biomarkers between groups

**Fig. 1:**
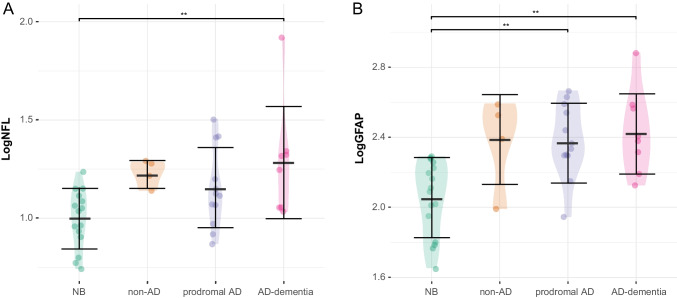
Plasma NFL and GFAP across groups. Values quoted in the y-axis indicate LogNfL and LogGFAP levels. Horizontal bars at the top indicate significant differences between groups. Horizontal bars superimposed on the violin plots indicate mean and SD. A. NB vs. AD-dementia (*p* = 0.004, *d* = 1.474). B. NB vs prodromal AD (*p* = 0.003, *d* = 1. 463); NB vs. AD-dementia (*p* = 0.002, *d* = 1.695)

CSF and blood biomarkers levels were different between groups: Aβ_42_ (*F* [3, 36] = 20.7, *p* < 0.001, η^2^ = 0.633), Aβ_42_/Aβ_40_ (*F* [3, 36] = 20.7, *p* < 0.001, η^2^ = 0.740), p-tau (*F* [3, 36] = 8.63, *p* < 0.001, η^2^ = 0.418), t-tau (*F* [3, 36] = 8.87, *p* < 0.001, η^2^ = 0.425) and LogGFAP (*F* [3, 36] = 7.65, *p* < 0.001, η^2^ = 0.389). As expected, post-hoc analysis showed that Aβ_42_ concentration and Aβ_42_/Aβ_40_ ratio were higher in NB compared to prodromal AD (*p* < 0.001, *d* = 1.901; *p* < 0.001, *d* = 3.330, respectively) and AD with dementia (*p* < 0.001, *d* = 2.39; *p* < 0.001, *d* = 3.269, respectively) as well as in non-AD compared to prodromal AD (*p* < 0.001, *d* = 3.193; *p* < 0.001, *d* = 2.706, respectively) and AD with dementia (*p* < 0.001, *d* = 3.684; *p* < 0.001, *d* = 2.645, respectively). On the opposite, p-tau and t-tau were lower in NB compared to prodromal AD (*p* < 0.001, *d* = 1.689; *p* < 0.001, *d* = 1.658, respectively) and AD with dementia (*p* = 0.002, d 0 1.731; *p* < 0.001, *d* = 1.812, respectively). There were no differences in p-tau and t-tau between non-AD, prodromal AD and AD dementia. LogGFAP was lower in NB compared to prodromal AD (p = 0.003, *d* = 1.463) and AD with dementia (*p* = 0.002, *d* = 1.695). LogNfL was lower in NB patients than in AD-dementia (*p* = 0.004, *d* = 1.474) (Fig. 1). There were no differences in CSF and plasma biomarkers among non-AD, prodromal AD and AD-dementia. There were no differences in plasma NfL and GFAP concentration between males and females (*p* = 0.737, η^2^ = 0.003; *p* = 0.64, η^2^ = 0.006, respectively) or between *APOE* ε4 + and *APOE* ε4- (*p* = 0.908, η^2^ < 0.001; *p* = 0.376, η^2^ = 0.023, respectively) (Table 2).

### Accuracy of plasma NfL and GFAP in predicting AD

**Fig. 2:**
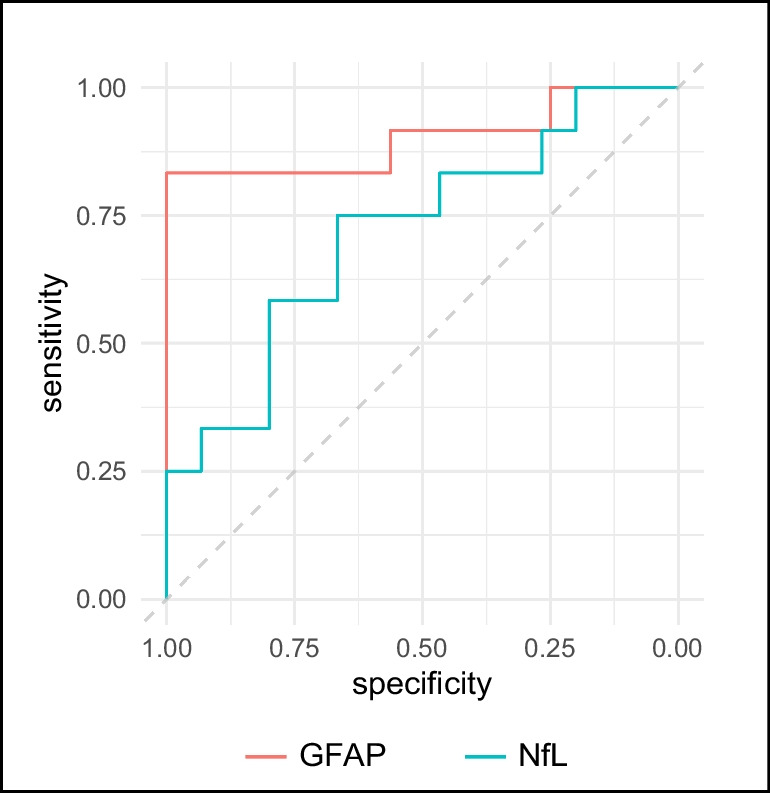
ROC curves for accuracy of NfL and GFAP in distinguishing NB and prodromal AD

We aimed to estimate the performances of plasma NfL and GFAP in differentiating patients with SCD and MCI who were carriers of positive AD biomarkers from patients who were not. Therefore, for this analysis we considered patients in the NB group and prodromal AD patients. We did not consider non-AD, due to the small number of patients, or AD-dementia patients, as we were not interested in differentiating patients with full-blown dementia from patients with SCD or MCI. NfL showed a fair accuracy (AUC = 0.718, accuracy = 70.37% [95% C.I. = 53.46: 87.28], sensitivity = 75.00% [95% C.I. = 58.96: 91.04]), specificity = 66.67 [95% C.I. = 49.21: 84.13], PPV = 61.54% [95% C.I. = 42.84: 80.24], NPV = 76.92 [95% C.I. = 60.73: 93.12]) in differentiating between NB and prodromal AD, with a cut-off value of 11.65 pg/mL. GFAP showed an excellent accuracy in differentiating NB from prodromal AD (AUC = 0.901, accuracy = 85.71% [95% C.I. = 72.75: 98.67], sensitivity = 66.67 [95% C.I. = 49.21: 84.13], specificity = 100%, PPV = 100%, NPV = 80.00 [95% C.I. = 65.18: 94.82]) with a cut-off level of 198.13 pg/mL (Fig. 2).

## Discussion

Plasma GFAP was highly accurate in predicting AD in patients with subjective symptoms or objective signs of cognitive decline, in line with the emerging literature [17]. The mean plasma GFAP concentration in our sample was consistent with previous reports on larger samples, but higher compared to Cicognola et al. [41]. Cicognola et al. also suggested a lower cut-off value (44 pg/mL) than the one we identified, despite using the same analysis methods for plasma NfL and GFAP. On the other hand, our cut-off value is slightly lower than the one identified by Oeckl et al. [42] (245 pg/mL) to differentiate patients with AD from healthy controls. This discrepancy may be explained by the fact that we considered patients with subjective cognitive decline (SCD) and mild cognitive impairment (MCI), while we did not include patients with AD dementia in the accuracy analysis.

Plasma NfL showed fair accuracy in discriminating between patients with normal biomarkers and patients with prodromal AD. These results are consistent with previous studies that indicated blood GFAP as more accurate than blood NfL. Nevertheless, in a previous study [43], we demonstrated that plasma NfL concentration was higher in patients with AD pathology (defined as A + /T + /N + or A + /T + /N- according to Jack et al. [2]) compared to patients with normal AD biomarkers and isolated Aβ pathology. We also showed that NfL concentrations were similar between patients with normal AD biomarkers and patients with isolated Aβ pathology. Therefore, based on our results, we can speculate that GFAP and NfL provide different information regarding the Alzheimer's continuum. GFAP seems to predict the presence of Aβ pathology regardless of tauopathy and neurodegeneration, while NfL might be more accurate in discriminating patients who also developed tauopathy, as supported by other authors [44]. This might also reflect previous findings showing that reactive astrocytosis is a very early process associated with Alzheimer’s pathology, preceding both amyloid plaque deposition and neurodegeneration [45, 46]. Consequently, we might suggest different roles for GFAP and NfL, with GFAP being more informative in the earlies stages of the disease and NfL being more useful in monitoring the progression of Alzheimer's pathological changes to Alzheimer's disease.

The main limitation of our study is the small sample size, particularly when we split the sample according to the ATN classification. Another limitation is that we did not include a sample of healthy control individuals. Additionally, being a single-center study, there may be biases related to assessment and diagnosis procedures. However, we would like to highlight some novelties of our work that may provide useful evidence for both clinical practice and AD research. While many studies have demonstrated the potential of NfL and GFAP, most of them have focused on predicting progression from MCI to dementia [19, 47, 48], whereas only a few studies have investigated the pathologic substrate of MCI defined according to AD biomarkers [19, 42, 44]. As the definition of AD has shifted from a purely clinical entity to a clinic-biological construct based on biomarker profiles, understanding the relationship between blood-based biomarkers and CSF biomarker profiles can have significant clinical implications for early AD diagnosis. Thus, we considered the presence of AD pathology as the outcome, biologically defined through CSF biomarkers, which is a strength of our study. Additionally, unlike previous larger studies that classified patients based on Aβ pathology [18, 19, 44, 47], we also considered tau pathology and neurodegeneration biomarkers, providing a comprehensive evaluation of plasma NfL and GFAP concentrations associated with different biomarker profiles. This is relevant as each biomarker profile has been associated with a different risk of progression to dementia. Moreover, only a few studies have included patients with SCD in their investigations of NfL, and we are aware of only one other study that assessed GFAP in SCD patients. SCD represents a clearly defined clinical entity with a higher risk of AD and progression to dementia compared to cognitively healthy individuals [49–52]. Based on this evidence, the National Institute of Aging-Alzheimer’s Association (NIA-AA) included SCD as the first manifestation of the symptomatic stages of AD preceding MCI. This distinction is relevant for the future application of blood-based biomarkers in clinical practice, particularly as disease-modifying treatments become available. It is widely understood that DMTs should be administered at the earliest stages of the disease to halt the pathological process before neurodegeneration begins [53]. However, general population screening may lead to an unacceptable number of false positive results and subsequent costs. In this perspective, patients with SCD represent an optimal selected population to be screened for prodromal AD. Our results provide one of the first pieces of evidence regarding plasma GFAP in this group of patients.

In conclusion, our work offers further insights into the utility of blood-based biomarkers in the prodromal phase of AD. Specifically, our results support the use of blood-based biomarkers in predicting Alzheimer’s pathology in patients with SCD and MCI, which represents a promising tool for biomarker assessment. This tool can also be applied at the primary care level to assist clinicians in determining the most appropriate and personalized assessment pathway for each patient.

## Data Availability

The datasets used and/or analyzed during the current study are available from the corresponding author on reasonable request.
